# A Peculiar Tracheoesophageal Fistula Caused by a Plastic Foreign Body

**DOI:** 10.1155/crot/3983529

**Published:** 2025-02-02

**Authors:** Chao Chen, Dingyuan Dai, Yu Guo, Chen Sun, Qi Li

**Affiliations:** Department of ENT, Children's Hospital of Nanjing Medical University, Nanjing, Jiangsu, China

**Keywords:** case report, pediatric foreign body aspiration, tracheoesophageal fistula

## Abstract

The occurrence of tracheoesophageal fistula (TEF) in children is commonly attributed to the presence of foreign bodies. This paper presents a unique case admitted to the Children's Hospital of Nanjing Medical University on August 9, 2022, where a plastic fragment resembling a doll's eye was identified as the precipitating factor. The report investigates the unusual trajectory of this case—examining the pathway through which the foreign body became lodged in the trachea, followed by the subsequent development of TEF—and provides valuable insights into its pathogenesis and clinical significance.

## 1. Introduction

Tracheoesophageal fistula (TEF) is a pathological fistula formed between the trachea and the esophagus. Based on its etiology, it can be categorized as congenital or acquired. Congenital TEF is typically associated with esophageal atresia and embryonic developmental anomalies, whereas acquired TEF can be caused by trauma, tumors, foreign bodies, or iatrogenic injuries [1]. Although acquired TEFs commonly originate from malignant tumors of the esophagus and lungs in adults, they are not the primary consideration when children present with TEF. In children, foreign bodies are the primary precipitating factor for TEF formation. With the diversification of electronic products in modern society, button batteries, due to their unique size and shape, are easily accessible to young children and can be ingested, subsequently lodging in the esophagus. Eventually, through chemical reactions, these foreign bodies can lead to serious injuries such as TEF [2]. Additionally, toys and other items commonly handled by children are often made of plastic [3], making plastic materials a common factor in pediatric airway and esophageal foreign body cases. This case report describes a rare instance of TEF originating from a foreign body resembling a plastic piece of a doll's eye. Upon examination, the child's esophageal mucosa was intact, and the foreign body was found in the trachea. Although conventional thinking suggests that such foreign bodies may be accidently ingested by children and become lodged in the airway, leading to TEF, intraoperatively, the diameter of the foreign body relative to the child's glottic diameter was not small, and it could not be smoothly extracted through the glottis. Therefore, through a retrospective analysis of this case, we investigated whether the foreign body was lodged in the trachea through the glottis and summarized the pathogenesis and clinical strategies for this TEF.

## 2. Case Presentation

The child of a patient, aged 4 years and 10 months, was admitted to the hospital on August 9, 2022, due to “chronic dyspnea lasting 2 years.” During this period, the child experienced no evident triggers for respiratory distress. During inhalation, the severity increased, accompanied by intermittent coughing and occasional production of yellow phlegm. Exacerbated by colds. The history of foreign body aspiration and swallowing is unclear, and there are no other distinctive circumstances. Previous treatments for “laryngitis” proved ineffective, prompting the patient to seek further medical attention at our hospital.

**Figure 1:**
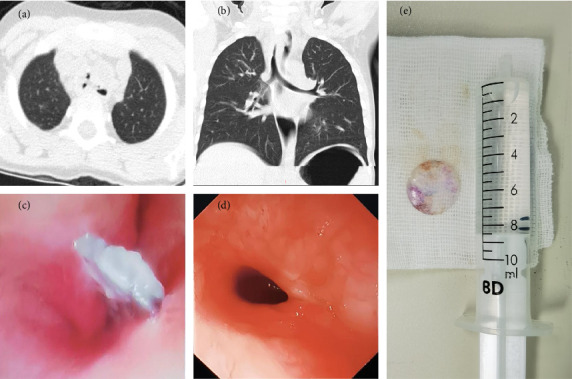
(a–b) Chest CT reveals abnormal density in the trachea and esophagus at the T1-2 level, accompanied by anterior left encapsulated gas and fluid collection, and foreign body with perforation. (c) Fiberoptic bronchoscopy reveals a foreign body at the mid-tracheal level with associated secretions. (d) Gastroscopy reveals smooth mucosa throughout the entire esophagus. (e) The foreign body is a round transparent plastic sheet, resembling a doll's eye, with a diameter of approximately 15 mm.

An emergency chest spiral CT scan (Figure 1(a), 1(b)) revealed abnormal density shadows in the trachea and esophagus at the T1-2 level, accompanied by encapsulated gas and fluid accumulation in the left anterior region, and a foreign body with perforation. Subsequently, the patient was diagnosed with an intratracheal foreign body and TEF and was admitted to the hospital for further treatment.

Upon admission, clinical examination revealed no cyanosis of the lip mucosa, a centered trachea, and symmetrical lung sounds without evident wet rales. A series of specialized examinations were planned, including fiberoptic bronchoscopy, gastroscopy, and rigid bronchoscopy, culminating in a tracheostomy for foreign body removal, scheduled for August 10, 2022.

**Figure 2:**
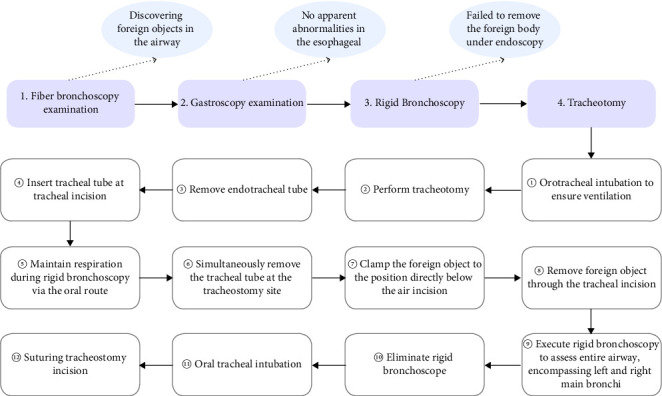
The key steps of surgery.

During the procedure under general anesthesia, a fiberoptic bronchoscopy through a laryngeal mask revealed a foreign body in the mid-trachea with adherent secretions (Figure 1(c)). After the removal of the laryngeal mask, a gastroscopy through the oral route showed a smooth esophageal mucosa without abnormalities (Figure 1(d)). A rigid bronchoscopy then confirmed the presence of a foreign body in the main trachea. Despite multiple attempts, the foreign body could not be maneuvered past the glottis, necessitating a tracheotomy for extraction. Therefore, we performed a transverse incision for tracheostomy and removed the foreign body using tracheal foreign body forceps through the tracheal incision. The retrieved foreign body was identified as a circular, transparent plastic sheet (Figure 1(e)). Exploration of the left and right main bronchi revealed no additional foreign bodies (The key steps of the surgery are shown in Figure 2).

**Figure 3:**
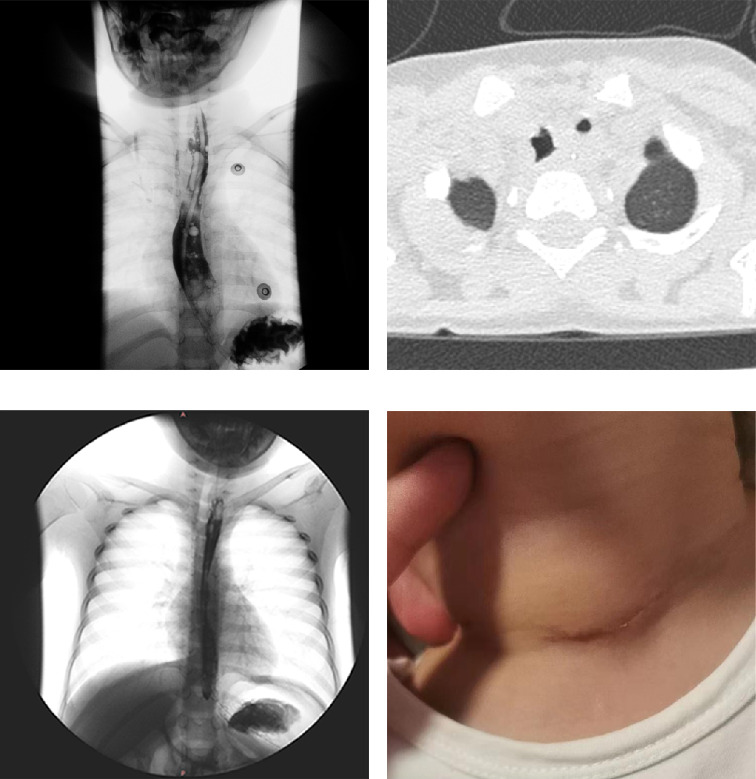
(a) A possible fistula between the trachea and esophagus at the T1-T2 level under esophageal iodine contrast imaging. (b) Re-evaluation of the chest CT scan demonstrates significant improvement in the pulmonary inflammation, no foreign bodies visualized within the trachea and esophagus, and the perforation has healed significantly compared to before. (c) Esophagography shows significant improvement in the tracheoesophageal fistula, with most of it healed compared to before. (d) The well-healed wound at the tracheostomy site in the pediatric patient.

Postoperatively, the patient was transferred to the SICU for mechanical ventilation and received anti-infection therapy, systemic intravenous nutritional support, and respiratory monitoring. The tracheal intubation was successfully removed on August 15, 2022. Despite a complication of bronchopneumonia, the patient responded well to anti-infection, anti-inflammatory, and antispasmodic treatments, alongside respiratory and nasogastric nutrition management. Esophagography performed on August 17, 2022, suggested a TEF (Figure 3(a)). The patient exhibited significant clinical improvement, including the resolution of asthma and cough symptoms, successful nasal feeding, and good healing of the neck incision. The patient was discharged on August 22, 2022, with recommendations for ongoing anti-infection treatment, nasogastric feeding, and nutritional support. Follow-up chest CT and esophagography in early September confirmed the healing of the TEF, and the patient progressively transitioned to a normal diet (Figures 3(b), 3(c)). By September 14, 2022, the patient had fully recovered, the drainage skin was removed, and the nasogastric tube was withdrawn, returning to a regular diet (Figure 3(d)).

## 3. Discussion

In this case, the patient's history of foreign body ingestion was indeterminate, and symptoms such as coughing and respiratory distress did not exhibit significant progressive exacerbation, thereby not prompting parental concern, leading to an initial lack of clarity regarding the condition. Preoperative chest CT and intraoperative bronchoscopy confirmed the presence of the foreign body in the airway, with granulation tissue noted at the site of foreign body retention, while esophageal mucosa appeared smooth and intact upon esophagoscopy. Hence, it was initially postulated that following accidental ingestion by the patient, the foreign body inadvertently passed through the glottis and became lodged in the trachea, subsequently resulting in a TEF. However, does the actual trajectory of the foreign body align with this hypothesis?

During the procedure, we found that the foreign body could not be retrieved via rigid bronchoscopy, as forcibly attempting to do so pose a significant risk of damaging the glottis. Hence, we ultimately opted for a tracheostomy to successfully extract it. Upon measurement, the diameter of the foreign body was approximately 15 mm, raising a new concern—whether the foreign body could have entered the airway through the glottis. A retrospective study analyzing 504 cases of emergency pediatric patients aged ≤ 4 years with normal neck *x*-rays positioned the glottis using computed tomography, calculating a mean anterior-posterior glottic diameter of 9.8 ± 1.5 mm [4]. Another study examining dissected specimens of newborns to infants up to 126 months of age found an average glottic length of 12.72 mm in the 42–126 months age group [5]. Considering the relevant medical history provided by the patient's parents, the child experienced respiratory distress without apparent cause 2 years ago, falling within the ≤ 4 years age group. Based on the medical history provided, the child experienced unexplained respiratory distress 2 years ago when they were under 4 years old. At that age, foreign objects had a diameter larger than that of the glottis, making it unlikely for them to enter the trachea through the glottis. Even if the child were to ingest foreign objects after turning four, the diameter of the glottis would still be smaller than that of the foreign object, resulting in a relatively low probability of foreign objects passing through the glottis into the trachea. Additionally, the child's respiratory distress persisted for 2 years without significant progressive exacerbation, whereas a foreign body of such diameter passing through the glottis typically induces severe coughing and progressively worsening respiratory distress. Postoperative esophagography (Figure 3(a)) also suggested a pre-existing fistula between the esophagus and the trachea. Hence, we refute the initial assumption of the foreign body entering the airway through the glottis, instead proposing that it remained lodged in the esophagus after ingestion by the child.

Given the foreign body's resemblance to a doll's eye, its hard texture, and overall flat convex shape, it likely adhered to the esophagus at its flat part after ingestion, causing minimal irritation to the esophagus. Symptoms were not apparent until the formation of a significant TEF due to prolonged pressure within the esophagus causing the gradual passage of the foreign body through the trachea, leading to the formation of the TEF observed in this case. However, due to the extended course of the illness, the foreign body may have remained relatively quiescent, allowing gradual self-repair of the esophageal mucosa, resulting in no apparent abnormalities on esophagoscopy. Additionally, the fistula may not have fully healed, with very small gaps still present, which could not be accurately detected by the naked eye using esophagoscopy. Chest CT, with its higher resolution, and postoperative esophageal contrast imaging, where the contrast agent, due to its extremely low molecular weight, could better penetrate into the remaining fistula gaps, thus radiological examinations objectively detected the TEF. Furthermore, due to mild inflammation and a longer course, the child tolerated it well; hence, symptoms such as coughing, respiratory distress, and swallowing difficulties were not prominent. Therefore, considering the aforementioned evidence, we ultimately conclude that in this case, the foreign body traversed from the esophagus into the trachea.

For TEF caused by foreign bodies, the initial step involves rapid identification of the foreign body within the trachea or esophagus through a combination of imaging studies and clinical symptom assessment. In the presented case, the pediatric patient's chest CT scans preliminarily indicated the presence of a tracheal foreign body, accompanied by exacerbated inspiratory breathing difficulties compared to previous instances, necessitating prompt surgical intervention for foreign body removal [6]. Bronchoscopy stands as the standard therapeutic approach for confirming and extracting tracheobronchial foreign bodies in children [7]. Rigid bronchoscopy remains the preferred method for removing tracheal foreign bodies in children, typically performed under general anesthesia [8–10]. Given the shared use of the airway by surgeons and anesthesiologists, an experienced team is essential to ensure adequate ventilation and oxygenation of the pediatric patient during surgery, which is crucial for the smooth progression of the procedure. The seamless coordination between surgical maneuvers, including tracheotomy-guided tracheal intubation, removal of the existing glottic tube, simultaneous maintenance of the patient's respiration, and foreign body extraction, underscores the significance of the medical team's proficiency and synergy.

Following the removal of the foreign body, the diagnosis and treatment of TEF require personalized management. Typical symptoms of TEF include choking after eating, severe coughing, feeding difficulties, and uncontrollable pneumonia [11]. However, when the foreign body remains lodged in the esophagus and has not completely formed a TEF, atypical symptoms such as intermittent respiratory distress and wheezing may occur, often overlooked or treated as other conditions like bronchial asthma [3]. Therefore, diagnosis of TEF can be made based on medical history, clinical presentation, imaging studies, and endoscopic examination, including but not limited to bronchoscopy, esophagoscopy, and esophagography, with endoscopy used to determine the location and size of the fistula [12]. Direct visualization using bronchoscopy and esophagoscopy is considered the gold standard for identifying TEF [13]. Once diagnosed, a treatment strategy must be decided upon. TEFs generally require surgery or endoscopic intervention once formed, but spontaneous closure does not necessitate surgical intervention. With adequate rest of the esophagus and rapid alleviation of surrounding inflammation, fistula closure can occur spontaneously [14]. Literature suggests that conservative treatment is advocated as the primary treatment choice as long as the patient's overall condition permits, and if signs of healing are observed, treatment should continue for no less than 1 month [15]. This approach helps avoid the invasiveness of surgery and the associated postoperative complications, while also being in a vigilant waiting period for self-closure, requiring close clinical monitoring of the patient's condition. Conservative treatment methods include esophageal rest, anti-reflux medication management, and nutritional supplementation via nasogastric, nasojejunal, or gastrostomy tubes, along with the use of antibiotics, clearance of airway secretions, and intravenous nutrition [11, 16].

However, the majority of acquired TEFs do not close spontaneously, especially those caused by common button batteries which can lead to alkaline corrosive injury to the gastrointestinal tract through isothermal hydrolysis reactions, ultimately resulting in secondary fistula perforation, often necessitating repair through open surgery or endoscopic procedures [17]. The choice of surgical intervention depends not only on whether the fistula can close on its own but also on the presence of severe complications, including aspiration pneumonia and bleeding [18]. After a vigilant observation waiting period, if there is no progress in fistula closure or if worsening of the condition occurs due to bleeding or respiratory distress, surgery should be promptly performed. Several factors determine the optimal outcome following surgical treatment of TEFs, including the timing and method of surgery, attention to important technical details, active use of antibiotics, and nutritional support [19]. Depending on the etiology, size, anatomical location of the fistula, and comorbidities of the patient, options for repair may include open or thoracoscopic surgery, as well as endoscopic therapy [20].

In this case, since the foreign body was a transparent plastic fragment with a flat shape adhering to the esophagus, causing minimal stimulation symptoms during the formation of the TEF, and with the esophagus receiving almost complete rest and surrounding inflammation alleviating, coupled with the relatively long course of the disease, esophagography indicated near spontaneous closure of the TEF. As there has been no recurrence, conservative treatment was ultimately administered to the child, obviating the need for further surgical intervention.

## 4. Conclusion

This case highlights the diversity of foreign bodies and the complexity of disease progression through analysis of the foreign body trajectory. Timely and accurate clinical diagnosis and management of TEF caused by foreign bodies are crucial and require comprehensive and individualized approaches. It also underscores the urgent need for otolaryngologists to possess solid theoretical knowledge and treatment skills. Additionally, for parents and caregivers, active education on child health and safety is imperative, encouraging exploration of the surrounding environment while emphasizing the importance of preventing potential risks and maintaining vigilant supervision.

## Data Availability

The image data used to support the findings of this study are included within the article.
